# Tobacco control policies in Italy: A time-trend analysis of smoking prevalence disparities, 2001-2023

**DOI:** 10.18332/tpc/225513

**Published:** 2026-10-21

**Authors:** Manuela Martella, Stefano Savigni, Valentina Ravazzani, Pietro Marraffa, Giacomo Scaioli, Maria Michela Gianino

**Affiliations:** 1 Department of Sciences of Public Health and PediatricsUniversity of TurinTurinItaly

**Keywords:** smoking trends, tobacco control policies, sociodemographic variables

## Abstract

**Introduction:**

Tobacco consumption remains a leading preventable cause of morbidity and mortality in Europe. In Italy, several public health initiatives have been implemented over the past two decades, including smoking bans, excise tax increases, and awareness campaigns. This study aims to examine time trends in smoking prevalence between 2001 and 2023, analyzing disparities by gender, age group, and education level.

**Methods:**

This secondary data analysis used nationally representative data from ISTAT, collected through periodic household surveys among individuals aged ≥14 years in Italy between 2001 and 2023. Time-trend analyses were conducted on the prevalence of smokers, non-smokers, and ex-smokers, including overall trends as well as stratified estimates by sex, age group (14-24, 25-44, 45-64, and ≥65 years), and education level (primary school, middle school, high school, and university degree).

**Results:**

Smokers' prevalence decreased by approximately 5% points over the study period (annual percent change, APC= -1.19; 95% CI: [-15.17; -0.85]; p<0.05). Among men, smoking declined across all age groups, with the greatest reductions in the youngest (aged 14-24 years, APC= -1.60) and oldest (aged ≥65 years, APC= -1.49) groups. Among women, smoking declined in younger age groups but increased among those aged ≥65 years. In terms of education level, smoking declined across all groups, particularly among individuals with primary school education. A notable increase was observed among university graduates in the final 3 years of the observation period.

**Conclusions:**

The findings highlight a long-term decline in smoking prevalence in Italy, with persistent disparities by gender, age, and education level. The recent increase observed in specific subgroups suggests a potentially changing pattern in tobacco use that warrants further monitoring and investigation.

## Introduction

Tobacco consumption remains a leading preventable cause of morbidity and mortality globally^1,2^. The European Region has the second-highest prevalence of tobacco use among both adults and adolescents, and tobacco consumption is linked to nearly 700000 deaths annually, with half of all smokers dying prematurely^1,2^. In response to the ongoing public health challenge posed by tobacco use, the European Union and its member states have enacted a range of tobacco control measures. Within this broader context, Italy has adopted a series of comprehensive tobacco control policies over the past two decades, also with a focus on reducing smoking among adolescents and young adults^3,4^. Key national interventions include the Law n. 3/2003 (Art. 51), also known as Sirchia Law, from the name of the promoting Ministry of Health^5^, which banned smoking in enclosed public spaces. The Law entered into force 2 years later and was further modified in 2013, extending the smoking ban to outdoor areas belonging to educational institutions, such as school courtyards, as well as to private vehicles when minors were on board^6^. In 2003, Italy took part in the WHO Framework Convention on Tobacco Control and signed the agreement concerning demand reduction (i.e. increasing taxes on tobacco products, implementing bans on tobacco advertising, creating smoke-free environments) and supply reduction (combating illegal trade of tobacco products, restricting access to minors)^7^. Finally, Italy adopted graphical health warnings on combustible cigarettes in 2016, covering 65% of the front and 65% of the back of the packages^8^. These national provisions have been periodically transposed from the European Community^8-11^. Complementary measures at the national level were further introduced, such as significant increases in cigarette prices driven by higher excise taxes, resulting in the near-doubling of the retail price of a cigarette pack in the last two decades^12,13^.

During the previous decade, the prevalence of smoking in Italy had declined significantly among both men and women, and the decline in smoking initiation was slightly more pronounced among women. Smoking initiation rates appeared highest among individuals from lower socioeconomic backgrounds, particularly those with only primary or secondary education, with the lowest rates of smoking cessation. Education has proven to be a stronger predictor of smoking behavior than occupation^14^. Tobacco consumption was historically more prevalent among men, the gender gap in rates has significantly narrowed in recent years^2^.

Previous Italian studies evaluated the immediate effect of the 2005 smoke-free law, as well as the medium-term impact (6 and 8 years after). However, while they agree on a prevalence decrease in the short-term, results over the following years were contrasting^15,16^. In this regard, to our knowledge, the possible long-term consequences of the smoke-free policies on prevalence and smoking cessation are not known. In addition, they focused only on the 2005 smoke-free law.

Few studies have assessed the effects of legislation tobacco control interventions in groups with different demographic and socioeconomic characteristics^17,18^, and only a handful of studies have assessed their impact, even differentiating the effects between short and long periods. In Italy, it has been found that among males with a low or high education level and females with a low education level, the smoke prevalence and cessation tend, after an initial reduction, to return to the pre-values smoke-free measure, while the free smoke measure was not associated with reduction among females with a high level of education^15^.

The current study aims to examine temporal trends in tobacco use in Italy over two decades following the implementation of major tobacco control policies, and to analyze whether changes in smoking prevalence and cessation differ according to gender, age, and education level. The study is based on nationally representative data, including individuals aged ≥14 years residing in Italy, surveyed annually between 2001 and 2023^19^.

## Methods

### Data source

This study is a secondary data analysis of publicly available data from the Italian National Institute of Statistics (ISTAT). The analysis included individuals aged ≥14 years living in Italy and was conducted on the proportions of smokers, non-smokers, and ex-smokers from 2001 to 2023, excluding 2004 due to data unavailability. Time-trend analyses were conducted on the proportions of smokers, non-smokers, and ex-smokers in Italy from 2001 to 2023, excluding 2004, due to unavailability. Data on the percentages of smokers, non-smokers, and ex-smokers were obtained from ISTAT. According to ISTAT definitions, smokers are individuals who report currently smoking, irrespective of frequency (daily or occasional) or quantity. Ex-smokers are defined as individuals who report a history of smoking, regardless of intensity or frequency, while non-smokers are those who report never having smoked. These data were collected through self-administered questionnaires as part of ISTAT’s periodic Multipurpose Survey on Households: Aspects of Daily Life - General Part^19^.

Data concerning the percentage of smokers, non-smokers, and ex-smokers were gathered from ISTAT, categorized by sex and age (14-24, 25-44, 45-64, and ≥65 years). Data related to the education level of smokers were classified into four categories (primary school, middle school, high school, and academic degree).

### Statistical analysis

Joinpoint regression analysis was applied to identify statistically significant changes in trends. The annual percent change (APC) was used as a summary measure to quantify the rate of change over time. The analysis included overall trends as well as stratified estimates by sex, age, and education level. According to the Joinpoint Regression methodology, the analysis initially fits a simple linear model to the data, then iteratively tests models with additional joinpoints. Each joinpoint indicates a statistically significant change in the trend’s slope. The final model is selected as the one with the fewest joinpoints that provides a statistically superior fit. Joinpoint analyses were conducted using the Joinpoint Regression Program (version 5.4.0). All statistical tests were two-sided, with a significance threshold set at p<0.05.

## Results

Overall, the baseline prevalence of smokers in Italy was approximately 24% of the population aged ≥14 years. Between 2001 and 2023, the proportion of smokers decreased by about 5% points, (APC= -1.19; 95% CI: [-15.17;-0.85]; p<0.05). A statistically significant joinpoint was identified between 2010 and 2019 (APC= -2.10; 95% CI: [-4.89; -1.51]; p<0.05). In the most recent 4 years of observation, a slight, though not statistically significant, increase in smoking prevalence was observed (APC=1.45; 95% CI: [-0.58; 5.71]; p>0.05). Correspondingly, the proportion of ex-smokers increased significantly over the same period (APC=0.52; 95% CI: [0.27; 0.79]; p<0.05), following an almost linear trend ranging from 20% to 25%. Two significant joinpoints were detected: the first between 2001 and 2006 (APC=2.51; 95% CI: [1.61; 3.90]; p<0.05), and the second between 2017 and 2023 (APC=0.91; 95% CI: [0.33 ; 2.82]; p<0.05). Among non-smokers, an overall upward trend was observed from 2001 to 2023 (APC=0.36; p<0.05). However, a significant joinpoint was noted during the final 4 years (2019–2023), during which a decline was recorded (APC= -0.68; 95% CI: [-16.46; -0.11]; p<0.05). Although not statistically significant, the proportion of non-smokers declined between 2001 and 2010, followed by a notable increase between 2010 and 2014 (Figure 1 and Table 1).

**Figure 1:**
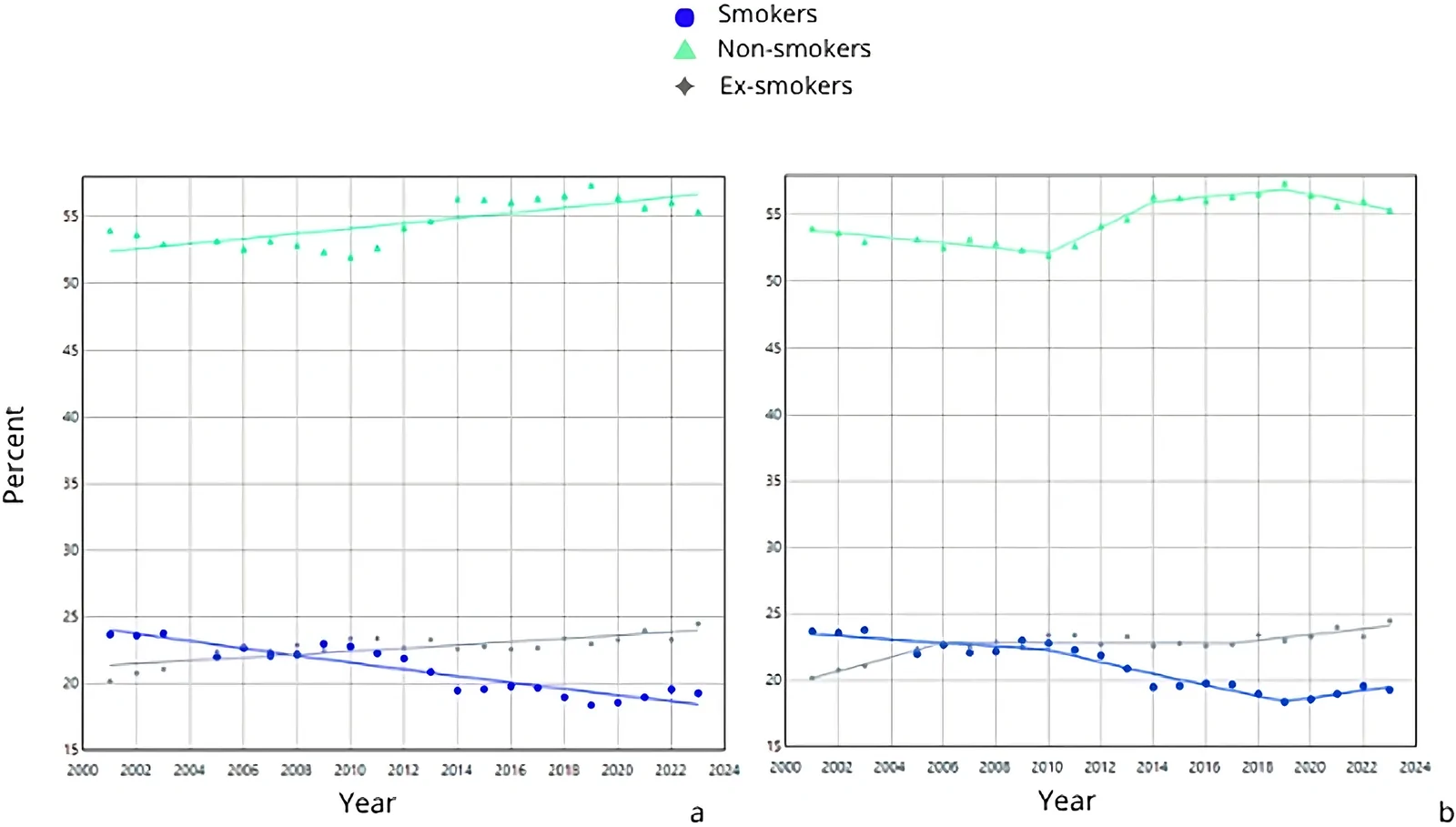
Trends in the prevalence of smokers, non-smokers, and ex-smokers: a) linear model - 0 joinpoint; b) selected joinpoint model; among individuals aged ≥14 years in Italy, based on nationally representative ISTAT survey data, 2001-2023

**Table 1:** Time-trend analysis of smoking status, overall, based on nationally representative ISTAT survey data, 2001-2023 (N about 45000-60000 individuals per year)

Smoking status	Timeframe	APC	95% **CI**	p
**Smokers**	2001-2023	-1.19*	[-15.17; -0.85]	<0.05
	2001-2010	-0.57	[-1.14; 1.56]	0.110
	2010-2019	-2.10*	[-4.90; -1.52]	<0.05
	2019-2023	1.45	[-0.58; 5.72]	0.228
**Non-smokers**	2001-2023	0.36*	[0.17; 0.54]	<0.05
	2001-2010	-0.35		
	2010-2014	1.79		
	2014-2019	0.33		
	2019-2023	-0.68*	[-1.65; -0.11]	<0.05
**Ex-smokers**	2001-2023	0.52*	[0.27; 0.79]	<0.05
	2001-2006	2.51*	[1.61; 3.90]	<0.05
	2006-2017	-0.02		
	2017-2023	0.91*	[0.33; 2.83]	<0.05

APC: annual percent change. JP: joinpoint(s). *Significance threshold set at p<0.05.

Age- and sex-specific trends revealed that smoking prevalence among women declined in younger age groups but increased among those aged ≥65 years. In contrast, smoking rates among women aged 45-64 years remained relatively stable over time. A significant decrease was observed for those aged 25-44 years between 2001 and 2018 (APC= -1.66; p<0.05), while two significant joinpoints were identified for those aged 45-64 years: an increase from 2001 to 2011 (APC=1.18; p<0.05) followed by a marked decrease from 2011 to 2014 (APC= -4.49; p<0.05). Among men, smoking prevalence significantly declined across all age groups from 2001 to 2023. The greatest reductions were observed among the youngest group (aged 14-24 years, APC= -1.60) and the elderly (aged ≥65 years, APC= -1.49). A significant decline was also reported for the age group of 25-44 years between 2010 and 2020, and for those aged ≥65 years between 2001 and 2017 (Figure 2 and Table 2).

**Figure 2:**
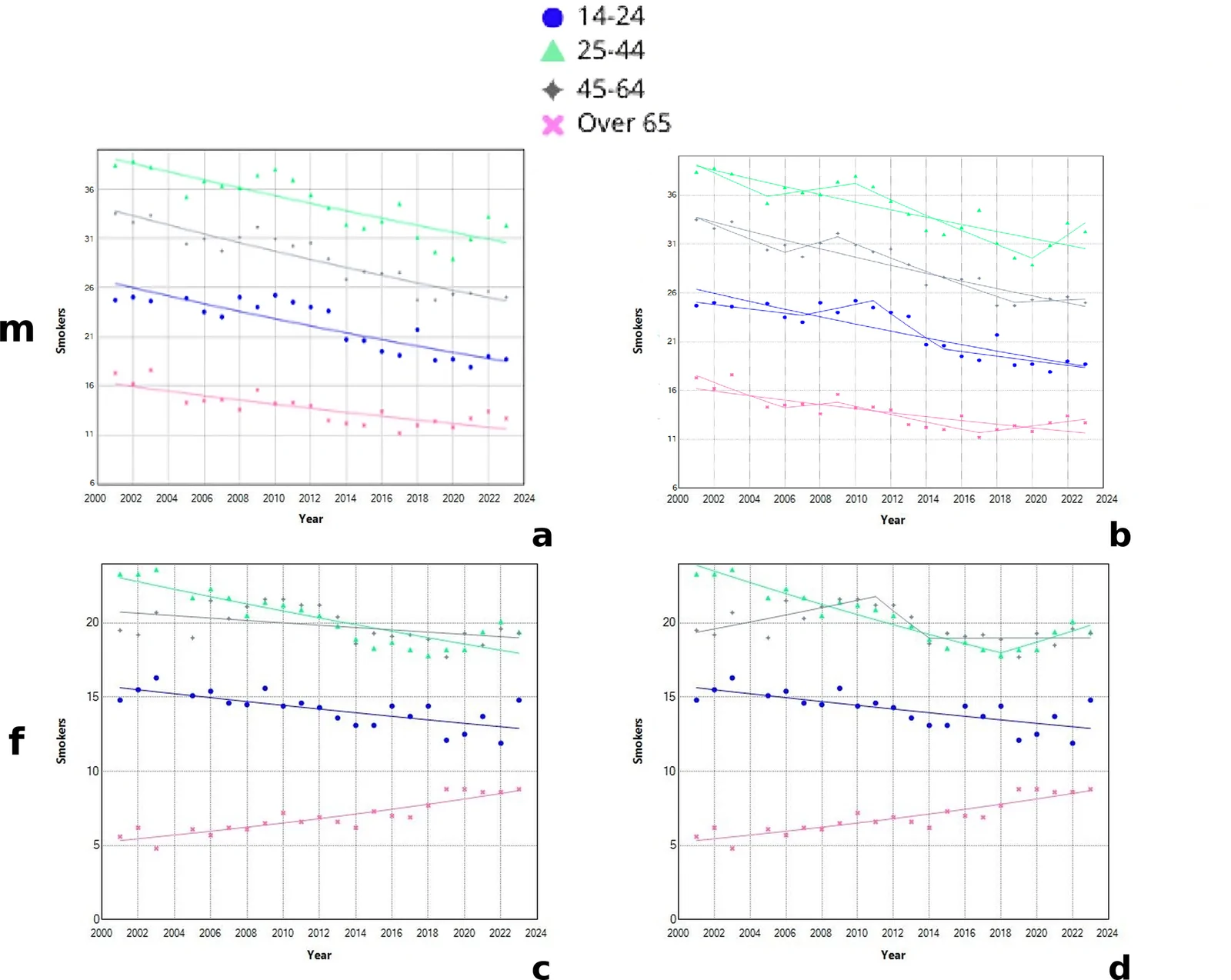
Trends of male (m) and female (f) smokers, stratified by age: a) linear model - 0 joinpoint; b) selected joinpoint model; among individuals aged ≥14 years in Italy, based on nationally representative ISTAT survey data, 2001-2023

**Table 2:** Time-trend analysis of smokers, stratified by sex, age and education level, based on nationally representative ISTAT survey data, 2001-2023 (N about 45000-60000 individuals per year)

**Females**		
**Age** (years)	**Timeframe**	**APC (linear**)
14-24	2001-2023	-0.87*
25-44	2001-2023	-1.13*
45-64	2001-2023	-0.4
≥65	2001-2023	2.24*
**Age** (years)	**Timeframe**	**APC (JP selected models**)
25-44	2001-2018	-1.66*
	2018-2023	1.99
45-64	2001-2011	1.18*
	2011-2014	-4.49*
	2014-2023	0.01
**Males**		
**Age** (years)	**Timeframe**	**APC (linear**)
14-24	2001-2023	-1.60*
25-44	2001-2023	-1.10*
45-64	2001-2023	-1.42*
≥65	2001-2023	-1.49*
**Age** (years)	**Timeframe**	**APC (JP selected models**)
14-24	2001-2012	-0.11
	2012-2015	-5.99
	2015-2023	-1.15
25-44	2001-2010	-0.36
	2010-2020	-2.08*
	2020-2023	3.69
≥65	2001-2017	-2.22*
	2017-2023	1.51
**Overall**		
**Education level**	**Timeframe**	**APC (linear**)
Primary school	2001-2023	-2.15*
Middle school	2001-2023	-1.43*
High school	2001-2023	-1.20*
Academic degree	2001-2023	-1.72*
**Education level**	**Timeframe**	**APC (JP selected models**)
Primary school	2001-2015	-2.86*
	2015-2023	-0.59
Middle school	2001-2007	-1.68*
	2007-2010	2.62*
	2010-2014	-4.69
	2014-2023	-0.83*
High school	2001-2010	-0.58
	2010-2019	-2.10*
	2019-2023	1.39
Academic degree	2001-2015	-1.36*
	2015-2018	-6.17*
	2018-2023	1.92*

APC: annual percent change. JP: joinpoint(s). *Significance threshold set at p<0.05.

In terms of education level, smoking prevalence declined across all categories, with the most pronounced reductions among individuals with either a primary school education or an academic degree. Among those with only primary education, a significant decrease was observed between 2001 and 2005. Conversely, individuals with a middle school education experienced a significant increase in smoking between 2007 and 2010, followed by a marked decline from 2010 to 2023. Among university graduates, smoking significantly decreased over the first 12 years (2001–2018), with the steepest decline occurring between 2015 and 2018. However, an increase was noted in the final 3 years of the observation period (Figure 3 and Table 2). Further details are given in the Supplementary file along with a STROBE checklist.

**Figure 3:**
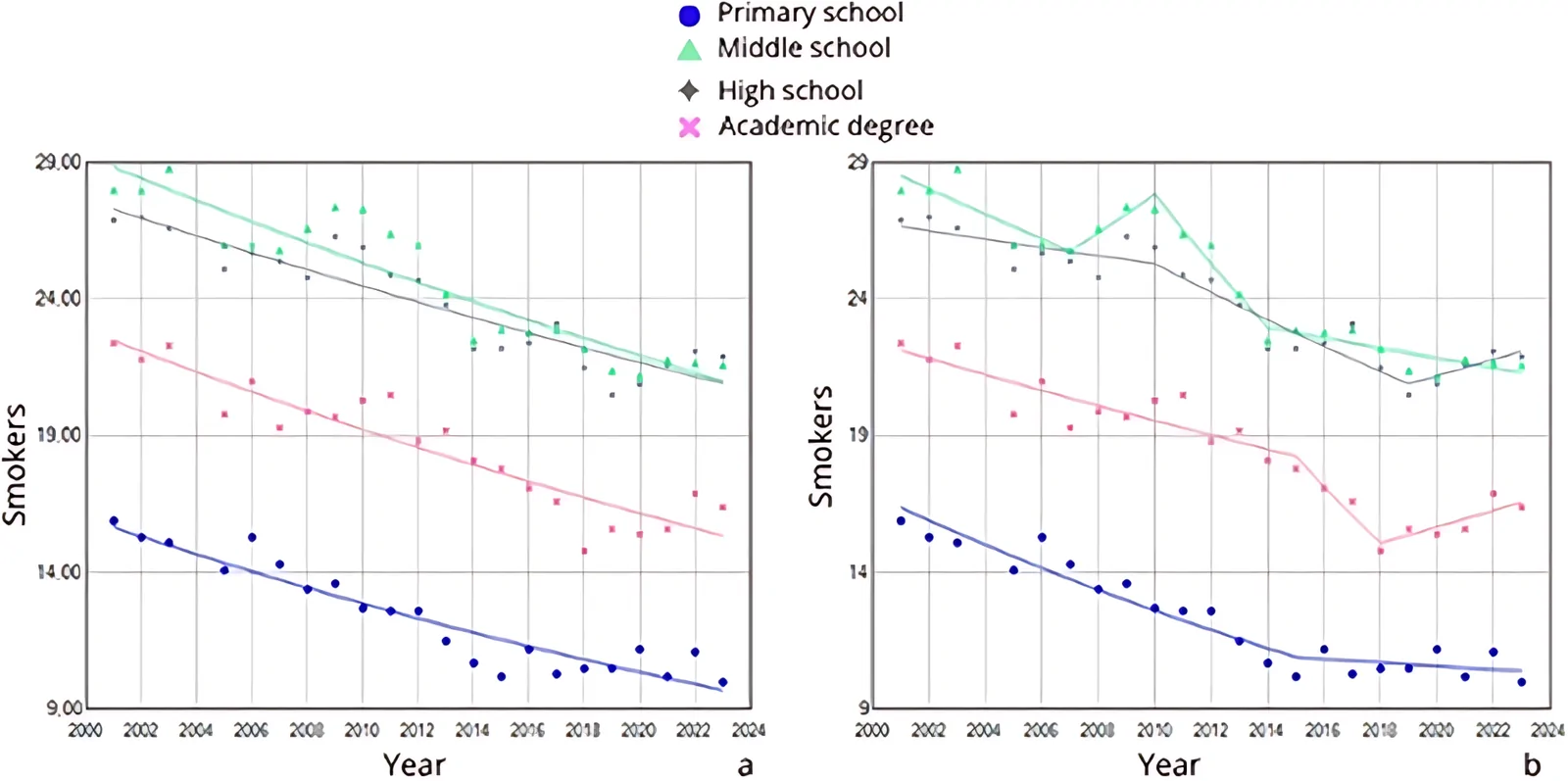
Trends of smokers by education level: a) linear model - 0 joinpoint; b) selected joinpoint model; among individuals aged ≥14 years in Italy, based on nationally representative ISTAT survey data, 2001-2023

## Discussion

The current research aims to evaluate changes in tobacco habits in Italy two decades after the introduction of the so-called Sirchia Law (Law No.3/2003, Art.51), which prohibited smoking in indoor public spaces and following other public measures. Additionally, the study seeks to identify behavioral differences in smoking patterns between men and women, as well as across age groups and levels of education level within the population.

The results show a significant decrease in smokers and a corresponding growth in ex-smokers and non-smokers between 2001 and 2023. Furthermore, they record no joinpoint related to the introduction of the ban law, nor the subsequent policies that took place in 2013 and 2016.

Our results are in line with a previous Italian study, which reported that between 2001 and 2013, an annual percentage change of -2.6%. However, the jointpoint clearly shows that the reduction is concentrated in the period 2010–2019, while in the subsequent period (2019-2023), the percentage of smoking increases, with a corresponding and significant decrease in non-smokers and ex-smokers. This trend is aligned with previous surveys^20,21^, which state that the downward trend in smoking prevalence has ceased and even suggest a possible increase in conventional smoking, and seems to align with a previous Italian study, which concludes that smoke-free policies may not have an effect on prevalence in long-term care^15^. The significant decrease in 2010–2019 may be explained by the synergistic effect of different smoking policies that may have influenced the success of the interventions in the short- to medium-term. The likelihood of long-term effects of tobacco control policies does not even appear to be supported by the introduction of some new products, such as e-cigarettes. Recent studies on the effects of these new tobacco and nicotine products did not observe an increasing trend in smoking cessation rates among e-cigarette users from 2014 to 2021, suggesting that the changes in e-cigarette devices and liquids did not impact smoking cessation^22^. In fact, the use of these products appears to increase, rather than decrease, the likelihood of smoking conventional cigarettes^23^.

Focusing on gender-specific trends, the prevalence of smoking among men remained consistently higher than that among women throughout the entire observation period (2001-2023). Although smoking prevalence declined in both sexes, the reduction was less pronounced among women, with the exception of older women (aged ≥65 years), among whom smoking rates not only failed to decrease but actually increased over time. These results found support in previous literature. In recent years, the prevalence of tobacco use among men has consistently remained higher than among women^24^. Over time, declining smoking rates among males have been observed, whereas smoking prevalence among females has generally either shown only slight decreases or, in some cases, even increased^25-27^. Literature highlighted gender discrepancies in successful quit smoking attempts^28-30^, with more difficulties for women and highlighted that specific characteristics of older women smokers are determinants on smoking cessation; older smokers often have long smoking histories, marked by strong nicotine dependence, previous unsuccessful quitting attempts, and doubts about the benefits of quitting^31^ other than fears of post cessation consequences, such as weight gain and increased stress or negative affect^31^. Consequently, it is difficult to interpret this result in the Italian context without further analysis of the characteristics of Italian older women smokers.

Considering education level, our findings indicated a higher yet comparable prevalence of smoking among individuals with a middle or high level of education, and APC was slower in comparison to primary school. These results, even if they do not reflect the conventional inverse gradient typically observed in previous Italian research^5,26^, are consistent with a more recent Italian study^20^.

It is more difficult to justify the reversed trend among individuals with an academic degree between 2018 and 2023. Although our joinpoint analysis does not identify a unique post-2020 segment, it is possible to hypothesize that the COVID-19 pandemic and associated lockdowns played a role. As suggested by international literature^32,33^, the widespread adoption of remote work may have contributed to increased smoking in the absence of workplace restrictions, though this hypothesis requires further investigation with studies specifically designed to test structural changes around the 2020 period. Since remote work/work from home was predominantly accessible to white-collar workers and individuals with higher levels of education, it may explain the increase in smoking rates among graduates in Italy. This reversed trend seems to highlight that even the continuous increase in price of tobacco products has not influenced the prevalence of smokers with higher levels of education, in contrast to literature reporting that those with higher levels of education may be more sensitive to price^21^. However, current findings remain mixed or unclear and require further investigation^34,35^.

### Strengths and limitations

A key strength of the present study is the use of a nationally representative dataset spanning >20 years, which allows for a comprehensive analysis of smoking habits within the Italian population. Furthermore, stratification by age cohort, sex, and education level enabled a more nuanced evaluation of this ongoing phenomenon, with a particular focus on sociodemographic aspects.

A limitation of the study is that the analysis was conducted at the national level, and the regional or macro-area (North, Center, South, and Islands) distribution was not analyzed. An analysis carried out within the national context may highlight particular differences that increase understanding of the factors that influence smoking cessation^25^. However, the state-wide level provides a better view to understand the possible impact of tobacco control policies. Also, the use of self-reported data may introduce information bias and potential misclassification of smoking status. Furthermore, as this is a descriptive time-trend analysis, it is not possible to establish direct causal relationships between the implementation of specific tobacco control policies and the observed changes in prevalence. Finally, while the dataset is nationally representative, the findings are specific to the Italian population and may not be fully generalizable to other contexts with different regulatory frameworks^36^.

## Conclusions

This study provides evidence on the prevalence trends of current, ex, and never smokers in Italy. While a long-term decline is evident, the slowdown observed two decades after the initial smoking ban suggests that existing measures may need to be reinforced or updated. Due to the descriptive nature of this study, we cannot formally conclude that policies have lost effectiveness; however, the data suggest the need for additional studies to obtain sufficient evidence on the long-term impact of tobacco control interventions.

The results suggest that it is essential to improve existing containment policies and developing new, targeted interventions and to prevent smoking initiation. Such efforts could include preventive multifaceted approaches that address individual, social, and environmental factors through targeted programs and initiatives. In addition to such initiatives, smoking cessation interventions, particularly those tailored by age and gender, could also be effective in addressing the increasing trend observed among older women in the Italian population. Certainly, although some evidence seems to deny it, to avoid the shift from conventional smoking to new products, the implementation of new policies is recommended. The major concern is currently the lack of funding for tobacco control, since no country in the EU, except Iceland, meets the recommended spending level of 2€ per capita. According to the Tobacco Control Scale^36^, scores related to spending remain extremely low, and several countries have experienced further reduction in funding, above all in 2020, due to the impact of the COVID-19 pandemic. Despite this situation, interventions are essential to reduce the burden of tobacco-related disease and protect public health.
