# Supplementary material for: Tobacco control policies in Italy: A time-trend analysis of smoking prevalence disparities, 2001-2023
Source: Tob Prev Cessat. 2026 Oct 21;12:10.18332/tpc/225513. doi: 10.18332/tpc/225513 (PMC13687674; doi:10.18332/tpc/225513)
Supplement: Supplementary file 1 [file TPC-12-66-s001.pdf]

TOBACCO PREVENTION AND CESSATION

Supplementary file

© 2026 Martella M. et al.

DOI:
10.18332/tpc/225513

The content has been provided by the author(s) and has not been reviewed, verified, or endorsed by European Publishing. It may not have undergone peer review. The views, opinions, and recommendations expressed are solely those of the author(s) and do not necessarily reflect the position of European Publishing. European Publishing accepts no responsibility or liability for any consequences arising from the use of, or reliance on, this content.

Table S1. Unadjusted annual prevalence (%) of smoking status (smokers, non-smokers, and ex-smokers) in Italy (2001–2023), based on nationally representative ISTAT survey data (N ≈ 45000–60000 individuals per year)

Year	Smokers (%)	Non-smokers (%)	Ex-smokers (%)
2001	23.7	54.0	20.2
2002	23.6	53.7	20.8
2003	23.8	53.0	21.1
2005	22.0	53.2	22.4
2006	22.7	52.6	22.8
2007	22.1	53.2	22.5
2008	22.2	52.9	22.9
2009	23.0	52.4	22.5
2010	22.8	52.0	23.4
2011	22.3	52.7	23.4
2012	21.9	54.2	22.7
2013	20.9	54.7	23.3
2014	19.5	56.4	22.6
2015	19.6	56.3	22.8
2016	19.8	56.1	22.6
2017	19.7	56.4	22.7
2018	19.0	56.6	23.4
2019	18.4	57.4	23.0
2020	18.6	56.5	23.3
2021	19.0	55.7	24.0
2022	19.6	56.1	23.3
2023	19.3	55.4	24.5

Note: Data for 2004 are not available.

STROBE Checklist

Tobacco control policies: time trends (2001-2023) and disparities in prevalence by gender, age, and educational level in Italy

Section/Topic	Item No.	Recommendation	Location in manuscript (page/section)
**Title and abstract**	1	(a) Indicate the study design with a commonly used term in the title or abstract<br>(b) Provide in the abstract an informative and balanced summary of what was done and what was found	Title (p.1): 'Tobacco control policies in Italy: a time-trend analysis (2001-2023) of smoking prevalence disparities by gender, age, and educational level'<br>Abstract (p.1): structured with Introduction, Methods, Results, Conclusions
**Introduction**<br>**Background/rationale**	2	Explain the scientific background and rationale for the investigation being reported	Introduction (pp.1-2): background on tobacco burden in Europe and Italy; Italian tobacco control laws (Sirchia Law 2003, extended 2013; graphical warnings 2016; excise tax increases); prior Italian studies (Federico 2012, Gualano 2014, Verlato 2014)
**Objectives**	3	State specific objectives, including any pre-specified hypotheses	Introduction (p.2): 'The current study aims to examine temporal trends in tobacco use in Italy over two decades following the implementation of major tobacco control policies, and to analyze whether changes in smoking prevalence and cessation differ according to gender, age group, and educational level'
**Methods**<br>**Study design**	4	Present key elements of study design early in the paper	Methods (p.2): secondary data analysis; time-trend analysis using Joinpoint regression on nationally representative ISTAT survey data (2001-2023)
**Setting**	5	Describe the setting, locations, and relevant dates, including periods of recruitment, exposure, follow-up, and data collection	Methods (p.2): Italy, national level; data 2001-2023 (excluding 2004 due to data unavailability); ISTAT Multipurpose Survey on Households: Aspects of Daily Life (annual)
**Participants**	6	(a) Cohort study: give eligibility criteria, sources and methods of selection<br>(b) Describe methods of follow-up<br>For matched studies: give matching criteria and number of exposed and unexposed	Methods (p.2): Italian population aged ≥14 years residing in Italy; aggregate data from ISTAT annual survey (N ≈ 45,000–60,000 per year); no individual-level follow-up (ecological/time-trend study); (b) & matching criteria: not applicable
**Variables**	7	Clearly define all outcomes, exposures, predictors, potential confounders, and effect modifiers; give diagnostic criteria if applicable	Methods (p.2): outcomes = annual % smokers, non-smokers, ex-smokers (per ISTAT definitions); stratified by sex (male/female), age groups (14-24, 25-44, 45-64, ≥65 years), educational level (primary school, middle school, high school, academic degree)
**Data sources/ measurement**	8	For each variable of interest, give sources of data and details of methods of assessment (measurement); describe comparability of assessment methods if there is more than one group	Methods (p.2): ISTAT SIQual database (ref. 19); self-administered questionnaires as part of ISTAT's periodic Multipurpose Survey on Households: Aspects of Daily Life – General Part; consistent methodology across years
**Bias**	9	Describe any efforts to address potential sources of bias	Strengths and limitations (pp.5-6): self-reported data may introduce information bias and potential misclassification of smoking status (acknowledged); national-level analysis without regional breakdown; no data on e-cigarettes or non-cigarette products

**Study size**	10	Explain how the study size was arrived at	Methods (p.2): entire Italian national population aged ≥14 years; 22 annual time points (2001-2023, excluding 2004 due to unavailability); N ≈ 45,000–60,000 individuals per year
**Quantitative variables**	11	Explain how quantitative variables were handled in the analyses; if applicable, describe which groupings were chosen and why	Methods (p.2): age groups (14-24, 25-44, 45-64, ≥65) and educational levels (4 categories: primary, middle, high school, academic degree) as pre-defined by ISTAT classification; annual prevalence proportions (%) used as continuous outcome
**Statistical methods**	12	(a) Describe all statistical methods, including those used to control for confounding<br>(b) Describe any methods used to examine subgroups and interactions<br>(c) Explain how missing data were addressed<br>(d) If applicable, describe analytical methods taking account of sampling strategy<br>(e) Describe any sensitivity analyses	Methods (p.2-3): (a) Joinpoint Regression Program v5.4.0; APC as summary measure; two-sided tests, p<0.05; (b) stratified analyses by sex, age group, educational level; (c) 2004 excluded due to data unavailability (no imputation); (d) aggregate nationally representative data; (e) no sensitivity analyses reported (descriptive study)
**Results**<br>**Participants**	13	(a) Report numbers of individuals at each stage of study<br>(b) Give reasons for non-participation at each stage<br>(c) Consider use of a flow diagram	Methods/Results (pp.2-3): (a) nationally representative aggregate data (N ≈ 45,000–60,000/year); 22 time points used; no individual-level exclusions; (b) 2004 excluded due to ISTAT data unavailability; (c) flow diagram not applicable for aggregate trend analysis
**Descriptive data**	14	(a) Give characteristics of study participants<br>(b) Indicate number of participants with missing data for each variable of interest<br>(c) Summarise follow-up time	Results (p.3): (a) baseline prevalence ≈24% smokers in 2001 (population aged ≥14); time series 2001-2023; Table 1 provides stratified trends by sex, age, education; (b) no missing data reported (aggregate data); (c) follow-up: 22 years (2001-2023, excl. 2004)
**Outcome data**	15	Report numbers of outcome events or summary measures	Results (pp.3-4): APC for smokers -1.19 (95% CI: -15.17, -0.85; p<0.05) overall; joinpoints identified 2010-2019 (APC -2.10); 2019-2023 APC +1.45 (ns); ex-smokers APC +0.52; non-smokers APC +0.36; see Table 1
**Main results**	16	(a) Give unadjusted estimates and, if applicable, confounder-adjusted estimates<br>(b) Report category boundaries when continuous variables were categorized<br>(c) If relevant, consider translating estimates of relative risk into absolute risk for a meaningful time period	Results (pp.3-4): (a) APC with 95% CI by sex, age group and educational level; Table 1; Figures 1-3; (b) age groups: 14-24, 25-44, 45-64, ≥65 (ISTAT pre-defined); (c) not applicable (APC used; absolute change ~5 pp decrease in smokers 2001-2023 reported)
**Other analyses**	17	Report other analyses done—e.g. subgroup, interaction, and sensitivity analyses	Results (pp.3-4): stratified analyses by sex (male/female panels; Figure 2: male panels a-b, female panels c-d), age group, and educational level (Figure 3); no interaction or sensitivity analyses (descriptive study)
**Discussion**<br>**Key results**	18	Summarise key results with reference to study objectives	Discussion (p.4): significant decline in smokers 2001-2023 (APC -1.19); reduction concentrated 2010-2019; recent upturn 2019-2023 (ns); increase in older women (≥65); reversed trend among university graduates 2018-2023; no joinpoint coinciding with 2003/2013/2016 policy introductions
**Limitations**	19	Discuss limitations of the study, taking into account sources of potential bias or imprecision; discuss both direction and magnitude of any potential bias	Strengths and limitations (pp.5-6): national-level data only (no regional/macro-area breakdown); no data on e-cigarettes or non-cigarette products; self-reported smoking

			status (potential information bias/misclassification); aggregate data (no individual-level analysis); cannot establish causal relationships between policies and trends
**Interpretation**	20	Give a cautious overall interpretation of results considering objectives, limitations, multiplicity of analyses, results from similar studies, and other relevant evidence	Discussion (pp.4-5): results consistent with Possenti 2025 (ref.20) and Federico 2012 (ref.15); long-term effectiveness of policies not confirmed; COVID-19/remote work as possible explanatory factor for university graduates trend (refs. 32-34); difficulty attributing trends solely to single policies; cautious interpretation throughout
**Generalisability**	21	Discuss the generalisability (external validity) of the study results	Strengths and limitations (p.5-6): nationally representative dataset over 20+ years (strength); results applicable to Italian context; regional heterogeneity not captured; findings may not be fully generalizable to other countries with different regulatory frameworks (ref.35)
**Other information**<br>**Funding**	22	Give the source of funding and the role of the funders for the present study and, if applicable, for the original study on which the present article is based	Statements (p.6): 'This research received no specific grant from any funding agency in the public, commercial, or not-for-profit sectors'

Note: STROBE = Strengthening the Reporting of Observational Studies in Epidemiology. This checklist refers to the final updated version of the manuscript (manuscript-she3.docx). Page numbers refer to the paginated manuscript body.
